# Chronic Heart Failure Rehabilitation: Diaphragm Training Needs More Attention

**DOI:** 10.3390/jcm14165624

**Published:** 2025-08-08

**Authors:** Bruno Bordoni, Bruno Morabito, Vincenzo Myftari, Andrea D’Amato, Paolo Severino

**Affiliations:** 1 Department of Cardiopneumology, Fondazione Don Carlo Gnocchi Onlus IRCCS, 20121 Milano, Italy; bordonibruno@hotmail.com; 2 Fondazione Policlinico Universitario Agostino Gemelli, Università Cattolica Del Sacro Cuore, 00168 Roma, Italy; brunomorabito84@gmail.com; 3 Department of Clinical, Internal, Anesthesiology and Cardiovascular Sciences, Sapienza University of Rome, Viale Del Policlinico 155, 00161 Rome, Italy; vincenzo.myftari@gmail.com

**Keywords:** heart failure, rehabilitation, physiotherapy, diaphragm, inspiratory muscle training, American Heart Association, American College of Cardiology, Heart Failure Society of America, European Society of Cardiology

## Abstract

Background: Chronic heart failure (HF) is a systemic condition in which the heart is unable to meet the body’s peripheral demands, leading to both acute and chronic functional decline, accompanied by high morbidity and mortality rates. A non-pharmacological, non-surgical standard approach to managing HF is cardiovascular rehabilitation, which is widely endorsed by international cardiology societies. This typically includes aerobic and anaerobic physical activity involving the peripheral skeletal muscles. However, international guidelines often overlook the clinical significance of the diaphragm and the role of inspiratory muscle training (IMT) in rehabilitation. The diaphragm plays a critical role not only in respiratory and cardiac function but also in supporting limb movements and overall physical performance. In patients with HF, diaphragmatic dysfunction contributes significantly to the symptoms they experience. Conclusions: This review highlights the need for a greater emphasis on incorporating IMT into the standard rehabilitation protocols for patients with HF, given its potential to improve both respiratory function and overall physical capacity.

## 1. Introduction

Chronic heart failure (HF) is a syndrome in which the cardiovascular system no longer responds adequately to the demands of the peripheral musculature and visceral tract under stress and, as the disease progresses, also at rest [1]. The global prevalence of HF is steadily increasing, making it a growing public health concern. This rise is largely attributed to an aging population, improved survival rates from acute cardiac events, and the increasing incidence of risk factors such as hypertension, diabetes, and obesity. In Asia, 31.89 million patients with heart failure (HF) were registered in 2019, with the incidence rates of HF in China (0.9% of the total HF population) and Thailand being 1032.84 and 900.90 per 100,000 people, respectively [2]. In Canada, an estimated 3.5% of the population suffers from HF [3]. Worldwide, approximately 64.3 million people (1.1–5.5% of the total population) suffer from HF, with similar percentages between genders. Over 80% of HF patients are aged 65 years or older and approximately 10% of people aged 65 years or older have HF [4,5]. Patients diagnosed with HF suffer from extreme disability (about 8.9% of the total), severe disability (about 30.3% of the total), or moderate/severe disability (about 53.3% of the total). A total of 70% of patients complain of chronic pain, and about half of patients suffer from anxiety and depression [4].

Projections indicate a steady increase in the incidence of HF, which will affect 95 million people worldwide by 2060; in America, HF is expected to increase by approximately 46% from 2012 to 2030 [1,6]. In general, around 550,000 new cases of chronic HF are recorded each year [7]. The survival rate of HF patients one year after their diagnosis is between 4% and 45%, depending on their nationality, age, comorbidities, and socioeconomic aspects [8].

The American Heart Association (AHA), American University of Cardiology (ACC), and Heart Failure Society of America (HFSA) guidelines for the management of HF have further defined the phenotypes of HF and categorized them into four categories based on left ventricular ejection fraction (LVEF) values. We can distinguish patients with a preserved LVEF (HFpEF with an EF of ≥50%), a mildly reduced EF (HFmrEF with an EF of 41–49%), a reduced LVEF (HFrEF with an EF of ≤40%), and an improved EF (HFimEF with an EF of ≤40% that improves by about ≥10%) [9,10].

The European Society of Cardiology (ESC) describes three types of HF, always based on the EF: HFpEF (LVEF ≥ 50%); HFrEF (LVEF ≤ 40%); and HFmrEF (LVEF 41–49%) [11].

The diagnosis of HFpEF is guided by the criteria outlined in specific clinical guidelines and includes, but is not limited to, the evaluation of LVEF values. The American guidelines, for example, recommend determining the levels of N-terminal pro-brain natriuretic peptide (NT-pro-BNP, ≥125 pg/mL) or brain natriuretic peptide (BNP, ≥35 pg/mL) regardless of the presence of a sinus rhythm or atrial fibrillation [12]. The ESC recommends that NT-pro-BNP and BNP levels should be assessed differently. In the case of a sinus rhythm, the same values apply as in the American guidelines, while in the case of atrial fibrillation, the physician must pay attention to NT-pro-BNP levels above 365 pg/mL and to BNP levels above 105 pg/mL [12].

Using HF categories, hospitalization and mortality data are more specific. Hospitalization for HFpEF reaches 198 per 1000 people per year, one in ten people aged 45 years are diagnosed with HFpEF, and the 5-year mortality from the time of diagnosis is 75% [13,14]. HFpEF is estimated to represent 19–55% of all HF cases, affecting elderly patients (over 65 years), with females accounting for a greater proportion than males [4,15]. On the other hand, the mortality rate for HFrEF is 53.5% five years from the time of diagnosis, with a rate of 50.6% and 47.2% for patients diagnosed with HFmrEF and HFpEF, respectively [10]. The hospitalization rate within one year is 31.9% for patients with HFrEF and 22% for patients diagnosed with HFmrEF [10]. Approximately 71% of patients with HFrEF are male [16].

Furthermore, a guideline-directed optimized medical therapy can ensure greater survival in patients with HF. The recommended treatment for HFrEF, according to international guidelines, includes angiotensin receptor-neprilysin inhibitors (ARNIs), angiotensin-converting enzyme inhibitors (ACE inhibitors), angiotensin receptor blockers (ARBs), beta-blockers, mineralocorticoid receptor antagonists (MRAs), and sodium-glucose co-transporter 2 inhibitors (SGLT2i) [17,18].

The reduction in mortality with the consumption of these drugs varies from 17% to 35%, and the risk of re-hospitalization is lowered to 21–41% [19]. There is an increasing number of therapeutic strategies that allow for the continuation of optimized therapy even during hyperkalemia, in order to maintain proper compliance and titration over time [20].

Patients with HFpEF, instead, may be treated with SGLT2 inhibitors, angiotensin receptor–neprilysin inhibitors (ARNIs) like sacubitril/valsartan, and non-steroidal mineralocorticoid receptor antagonists (nsMRAs). Additionally, glucagon-like peptide-1 (GLP-1) receptor agonists, such as semaglutide, may be considered in obese patients [21,22]. According to the AHA/ACC/HFSA guidelines, patients who fall into the category of HFimEF should continue to take drugs for patients with HFrEF indefinitely [23]. For patients with HFmrEF, the ESC/AHA/ACC/HFSA guidelines suggest the same treatment as that for patients with HFpEF, with reservations [17,18].

Another key non-pharmacological strategy involves structured physical activity, encompassing aerobic and anaerobic exercise, as well as respiratory muscle training. Extensive evidence indicates that regular physical activity and rehabilitation improve symptoms, delay rehospitalization, enhance quality of life, and reduce mortality in patients with HF. However, standardized guidelines specifying exercise regimens tailored to particular HF subtypes or severity levels are currently lacking [24]. Although current recommendations for respiratory muscle training or inspiratory muscle training (IMT) remain limited and inconsistent, greater emphasis should be placed on incorporating IMT into patients’ rehabilitation and exercise programs, given the essential systemic role of respiratory function [25,26]. The aim of this article is to explain the importance of the diaphragmatic muscle in the context of the training/rehabilitation of patients with HF.

## 2. Physical Rehabilitation in Chronic HF

### 2.1. Endurance Training

Aerobic activity/endurance training (ET) is a “must” in the rehabilitation process, despite being insufficiently used [6]. Failure to pursue rehabilitation training is the cause of approximately 45% of mortality and re-hospitalization in patients with HF [6]. The reasons for this are many, such as the patient living an excessive distance from the rehabilitation center and the patient’s economic status, as well as the clinical picture being too critical.

ET, which can be started in stable patients, involves large muscle groups used in a repetitive manner, including riding on an exercise bike, brisk walking or running on a treadmill, swimming, stair climbing, or lifting light weights while maintaining an aerobic regime [24]. The main goal is to improve exercise oxygen consumption (Vo2max); the latter value is predictive of exercise capacity and clinical outcome [6].

In the American guidelines, a patient is considered stable in the absence of cardiac events over a period of ≤6 weeks, whereas in the ESC guidelines, a patient is stable if they go approximately one month without symptoms or cardiovascular events, indicating they can undergo ET [27]. Despite the definition of stability, the guidelines recommend a rapid start to rehabilitation after their admission to hospital [3,28].

Before starting exercise training, patients must perform a cardiopulmonary exercise test (CPET) to determine subjective work thresholds [6,27]. However, the values derived from the CPET (VO2Max/peak heart rate) do not always reflect the patient’s cardiovascular and respiratory subjectivity. For example, patients with HFrEF might achieve a non-maximal VO2Max threshold (<85%); and patients with comorbidities that limit voluntary exercise would not be able to show their maximal capacity to utilize oxygen [6].

There are different ET modalities that should follow the FITT rules (frequency, intensity, time, and type of exercise), although there is no specific indication that is considered the gold standard [28]. Moderate-intensity continuous training (MICT) is well tolerated and safe for patients with HF, even for frail people with a low work capacity (<3 METs) [29]. According to the American guidelines, MICT should be at an intensity of 60–70% of the patient’s heart rate reserve (3–5 sessions per week; 40 min of exercise with a 10 min warm-up and a 10 min cool-down; 60 min total); the increase in ET is always gradual, both in terms of time and intensity [29].

According to the Canadian guidelines, MICT should be at an intensity of 65–85% of the patient’s heart rate reserve for 20–60 min and on several days per week [6]. The ESC recommends MICT at an intensity of 70–80% of the patient’s Vo2peak, 3–5 days per week, with 45–60 min per session and always with gradual increases [28].

High-intensity interval training (HIIT) is another ET approach. HIIT, in fact, consists of intense training interspersed with rest (completely interrupted or at a reduced intensity) and recovery. There are currently no guidelines that specify the correct form of HIIT, and to our knowledge, there is no consensus on the superiority of one modality over the other [6,27,29,30,31]. According to the American guidelines and the ESC, it is recommended to engage in 1–3 sessions per week, with an interval time of 1–2 min or 3–4 min, depending on the functional capacity of the patient with HF [27]. Though it always has a warm-up and cool-down period, HIIT training can vary, with four periods of high effort at 85–95% of the patient’s VO2peak (3–4 min or 30–60 s each), alternated with moderate effort at 50–70% of the patient’s VO2peak, each minutes or seconds long [30]. One session of HIIT should be a maximum of 35–45 min [3]. The patient can also perform low-intensity interval training, with series of 10–30 s and rests of 60–80 s, for a maximum duration of 10 min, at an intensity of 50% of their VO2peak [3]. In general, it is recommended to guide patients with HFrEF towards HIIT or low-intensity interval training [28,30].

### 2.2. Resistance Training

Another training modality is resistance training (RT) or strength training. RT can and should be included in the patient’s rehabilitation program approximately 2–4 weeks after the start of ET, avoiding expiratory apneas (the Valsalva maneuver) [27]. Moreover, RT should be combined with ET, with an average of 2–3 sessions per week and with an intensity that is always subjectively adapted to the person’s functional status, for a total of 30–60 min per week [6,27,28,32,33]. The load for RT is calculated based on the percentage of the maximal contraction (1RM) that is possible with a given load. Initially, a load reflecting <30% of the 1RM is used (low intensity), with 5–10 repetitions and with a rating of perceived exertion (RPE; 6–20 on Borg scale) of 10–12, and the number of sets is subjective [24,28]. As training progresses, one can progress to moderate intensity (30–60% of the 1RM) with an RPE of 11–13 and with more repetitions (8–12) and sets (not specified). High-intensity RT uses 80% of the 1RM, with an RPE of 13–16 and 8–15 repetitions, for 1–2 sets [24]. The ESC and American guidelines recommend moderate-intensity RT, in which the greatest cardiovascular benefits can be achieved, and a safe exercise regime, performing 8–10 exercises working the major muscle groups [28,33].

There is no specific evidence of RT regarding the types of HF. This is a grey area for guidelines and rehabilitation practice.

### 2.3. Benefits with Physical Activity

Physical activity offers multiple benefits to patients with heart failure, including an improved functional capacity (e.g., increased walking distance) and enhanced quality of life. Notably, patients who fail to demonstrate statistically significant improvements, regardless of their LVEF, are at a higher risk of mortality [6].

The combination of ET and RT improves VO2max by approximately 4–16% and chronotropic incompetence; a 6% increase in VO2max reduces all-cause mortality and hospitalizations by 5–8 in patients with HF [6,27,29,34].

Regular physical exercise reduces sympathetic nervous system activity and may positively influence post-infarction cardiac remodeling. It also enhances the body’s antioxidant capacity, lowers systemic inflammation, improves both the structure and function of the endothelium, and increases peripheral (skeletal muscle) oxygen extraction at rest and during exertion. Additionally, it promotes greater cardiac electrical stability and stimulates the development of the coronary collateral circulation [3,6,26,34].

Blood glucose levels improve at rest and during exercise and are better controlled [26]. LVEF values may increase, although the underlying mechanisms are not fully understood. Diastolic function, as reflected by a reduction in the E/e’ ratio and the prolongation of deceleration time (DT), may undergo favorable changes [34]. The increase in cardiac contractility mainly affects patients with HFpEF; moreover, there is no direct correlation between the improvement in VO2max and EF [34]. Some clinical changes detectable with ET and RT could be related to the phenotype of the patients in the conducted study, such as age, gender, baseline clinical values, anthropometric values, and comorbidities, as well as a lack of standardization [4,35]. Currently, there are no concrete data on the specificity of adaptation depending on the type of HF (Table 1).

## 3. Inspiratory Muscle Training (IMT)

In the American guidelines (AHA/ACC/HFSA), there are no recommended procedures for improving the functional capacity of the respiratory muscles [21]. In the ESC guidelines, we find some evidence based on the measurement of maximal inspiratory pressure (PImax) by spirometric testing [28]. These references are specific to HFrEF and suggest that training should start with a resistance of 30% of the patient’s PImax, reaching up to 60% of the patient’s subjective PImax. Resistance needs to be recalibrated approximately every week, with 3–5 sessions per week and 20–30 min per session; it is also recommended to add IMT with ET and RT [28]. However, it is not specified if breaks should be taken between breaths, whether the series within each session are planned, whether the patient must perform a single breathing cycle when training with resistance, and what possible symptoms should be ignored during IMT. The available research and reviews report numerous positive effects when IMT is added to the usual rehabilitation program (ET and RT), although great heterogeneity should be noted.

IMT increases patients’ PImax and VO2peak, increases the slope of minute ventilation/carbon dioxide production (VE/VCO2), reduces the feeling of dyspnea, increases the distance covered in the 6-min walk test (in patients with HFpEF), improves their quality of life, lowers lactate levels, and improves ventilation efficiency, especially in combination with HIIT [3,5,7,24,26,27,36]. The number of patients with HF who have undergone IMT in various studies is small [37]. It is suggested that IMT could be used as a strategy in patients with poor adherence or non-adherence to physical activity routines [36] (Table 2).

Another effect that seems to be associated with IMT is the enhancement of the baroreceptor reflex, accompanied by improved peripheral perfusion during exercise and a reduction in peripheral chemoreceptor sensitivity. Additionally, an increase in lower limb muscle strength has been observed [24,36]. IMT in combination with ET reduces the levels of NT-pro-BNP and C-reactin protein [3]. A rehabilitation program that included ET/RT/IMT appeared to offer better functional benefits in patients with HF (in terms of circulatory and respiratory function, according to the ARISTOS-HF study) compared to ET/IMT [38]. In a more recent study (2022), IMT (40% PImax; 5 sets with 10 repetitions and 1–2 min rest between sets; 30 min per session and 3 sessions per week for 6 weeks) was investigated in advanced heart failure (AHF, New York Heart Association, NYHA class III or IV, HFrEF) [39]. Patients with reduced EF values may benefit from IMT, e.g., through improvements in chronotropic, diastolic, and systolic blood pressure and blood saturation, as well as an increased walking distance and increased inspiratory power, potentially arising from optimized autonomic nervous system function [39]. Similar results were found in another study on ET/RT/IMT (30% PImax; seven sets with an unspecified number of repetitions; 3 min of work and 1 min of rest for a maximum of 30 min; three weekly sessions over 6 months) for HFrEF patients. This study had a lower number of patients, but also evaluated their quality of life, which improved [40]. IMT could be used in acutely hospitalized patients (HFrEF) with low resistance (30% PImax; 15 repetitions and two sets per day with 1 min rest between sets; 5 days per week; no standard duration for IMT is given) [41]. IMT in this type of patient increases their functionality as measured by the distance walked during the 2-min walking test in meters [40]. Moreover, IMT in HFrEF patients with a high level of resistance (70% PImax; seven sets for 2 min with 1 min of rest between sets; a total of 21 min of IMT and 7 min of rest; three sessions per week for 8 weeks) leads to several functional improvements, such as more balanced autonomic expression, less dyspnea, increased diaphragmatic thickness, and better arterial response (such as tone and vasodilation) [42] (Table 3).

**Figure 1:**
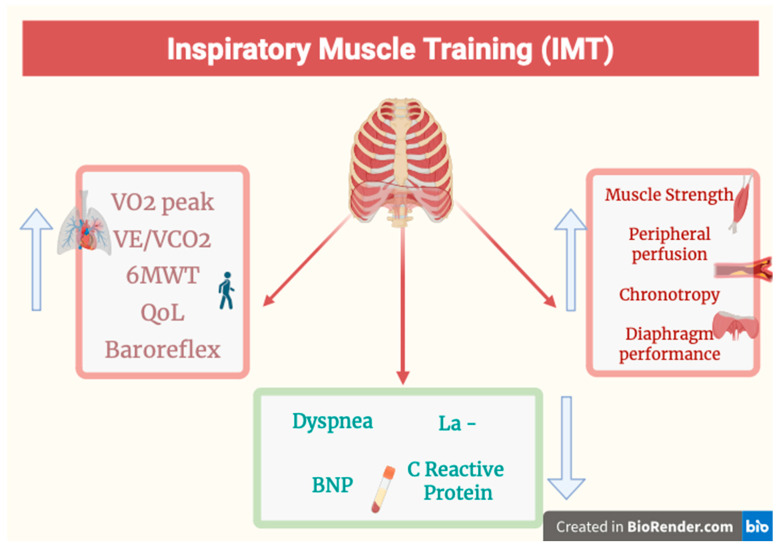
Summary of the benefits that can be obtained with diaphragm training (IMT) in patients with HF. IMT improves oxygen consumption under exertion (VO2peak), the minute ventilation/carbon dioxide production slope (VE/VCO2); increases the distance traveled inferred from the 6-min walk test (6MWT); increases the patient’s quality of life (QoL); increases autonomic responses (baroreflex); increases the strength and perfusion of peripheral muscles; improves cardiac chronotropy and the general function of the diaphragm. IMT decreases the values of blood lactate (LA-), N-terminal pro-brain natriuretic peptide (NT-pro-BNP), and inflammatory indices (C-reactine protein). Figure created with BioRender.com.

Despite many encouraging results, IMT is not yet considered fundamental for HF patients [15,25] (Figure 1).

## 4. Pathophysiological Rationale for IMT

In patients with chronic heart failure, skeletal muscle exhibits myopathic changes characterized by phenotypic, structural, and functional alterations. These changes adversely affect subjective symptoms such as fatigue and dyspnea and are associated with increased morbidity and mortality rates, regardless of the LVEF value [43]. The musculature is hypotrophic with a metabolic change towards a greater number of anaerobic (poorly functioning) fibers and reduced capillarization; these pathological adaptations promote an anaerobic metabolic environment even when exerting a small amount of effort, resulting in chronic afferent signaling (via type III and IV afferents) to the central nervous system. Peripheral information from skeletal musculature is processed by the central nervous system as constant fatigue, with an overactivation of the sympathetic system, vasoconstriction, a decreased VO2peak, an increased heart rate, and intolerance to effort, a condition known as “muscle hypothesis” (exaggerated mechano-metaboreflex). These myopathic alterations can be found in both HFrEF and HFpEF patients [43].

In this context, the diaphragm undergoes non-physiological adaptations faster than in the limb muscles [44]. There seems to be a degeneration of the synaptic plate with partial denervation and a reduction in force expression of 15–30%; in animal models, the contraction speed is reduced by 20–30%, which leads to a decrease in peak force (for example, when coughing or under stress) of 35–50% [44]. In animal models, an alteration in the excitation capacity of the muscle fiber is found, with a decline in the number of cross-bridges, titin (shock absorber protein), myosin heavy chain (MHC), and the movement capacity of the same protein, with a decrease in myosin ATPase activity [44]. In elderly patients with HF, a targeted decline in anaerobic fibers may occur [45]. A recent biopsy study of patients (21) who were not elderly highlighted a tendency towards an increase in red fibers compared to anaerobic fibers (with the opposite tendency in the limbs) [46].

In human models, myopathy leads to an increase in connective and adipose tissue within the diaphragm, which are signs of atrophy, with a decline in MIP of about 30% [45]. Diaphragmatic fatigue occurs earlier in patients with HF [47]. During an acute phase of decompensation, the diaphragm moves with a reduced excursion (measurable by ultrasound) and with an inverse relationship between the severity of symptoms (NYHA stage) and contractile capacity, as well as an inverse relationship between diaphragmatic movement and pulmonary artery systolic pressure [48]. The diaphragm presents with a decreased thickness (as shown via ultrasound) compared to healthy subjects [48]. A dysfunctional diaphragm in patients with HFpEF and HFrEF contributes to early fatigue, exercise intolerance (a decrease in peakVO2, which is related to vital capacity), and dyspnea [5,27,49].

Dyspnea and exercise intolerance are directly related to increased mortality, and a low PImax is related to symptom severity [7,15,40,50,51]. Furthermore, decreased diaphragm thickness is a prognostic factor. Diaphragm protein loss is related to decreased limb strength, a lower forced expiratory volume in one second (FEV1), and an increased mortality rate, blood BNP value, and frailty parameters [52]. The diaphragm does not play a marginal role in the clinical picture of patients with HF; rather, it plays a central role in both circulatory and respiratory function [52] (Table 4).

### 4.1. Role of the Diaphragm in Cardiac Function

The contraction/relaxation of the diaphragm allows for pressure fluctuations in the thorax, greater lung function, the movement of veins and lymphatic fluid, and the maintenance of adequate esophageal pressure [53,54]. Inspiration allows for the creation of an appropriate pressure gradient for venous return to the right atrium, with proper pressure in the right heart area (atrium and ventricle) achieved by increasing myocardial stretch (preload) and right stroke volume [55]. Inspiration influences the left ventricular afterload and the diastolic pressure gradient in the aorta. Expiration fills the left atrium and left ventricle, leading to a reduction in the aortic diastolic pressure gradient, and the left ventricular stroke volume increases [55]. The quantity/quality of cardiac output is highly dependent on the health of the diaphragm [55]. The diaphragm influences the pericardial pressures; they increase during exhalation and decrease during inhalation [55].

Pressure management, when the diaphragm is not dysfunctional, allows us to adequately modulate a patient’s hemodynamic values and baroreceptor responses, with an increase in the activity of the parasympathetic system (improving the chronotropic response) [56,57]. It is important to note that enhanced parasympathetic activity may contribute to the improvement of depressive and anxious symptoms in patients, whose emotional state has been shown to negatively impact both mortality and hospitalization rates [35,56,57].

### 4.2. Diaphragm and Muscular Strength

Patients with HF are subject to a high percentage of accidental falls for various reasons, such as cognitive decline, polypharmacological treatment, advanced age and frailty, an alteration of the autonomic system, arrhythmias, and various comorbidities [58,59,60].

One of the functions of the diaphragm is to improve one’s posture, stimulating during respiratory movement most of the proprioceptive receptors (exteroception and interoception), which send adequate afferents to the cortical and subcortical processing centers, passing through the spinal pathways (spino-solitary and spino-trigeminal pathways). The efferents return to the nucleus of the solitary tract to improve the response of the parasympathetic system, which is crucial for adequate postural coordination [53,61,62,63]. Furthermore, it is essential for the creation of abdominal and dorso-lumbar pressures for the stabilization of the spinal column during body movement [64].

To the authors’ knowledge, there is no research to evaluate the possible relationship between myopathic structural and functional alterations of the diaphragm and the percentages of accidental falls in this type of patient, nor the distinction between the different categories. In patients with HF, breathing is superficial and at a higher rate than in healthy subjects (muscle weakness); this more superficial breathing is not able to reduce the activity of the sympathetic system [65]. It seems that this reflex effect of breathing is more accentuated in women; with inspiration, the diastolic pressure increases, while with expiration, the systolic pressure varies only a little [66,67].

We know that elderly subjects engaging in IMT (~50% MIP) strengthens their postural balance, muscle and respiratory strength (peak inspiratory flow rate and inspiratory peak power), and overall coordination, compared to elderly subjects not undergoing IMT [68]. A recent systematic review reiterates that IMT in elderly subjects (50–75% MIP; 7 days/week) improves their balance, neuro-coordination, and trunk/limb muscle strength, as well as improves autonomic intervention in position changes (a better blood pressure response) [69].

On the other hand, another systematic review reports improvements in balance and motor function in elderly patients with different chronic pathologies (stroke, HF, chronic renal failure, diabetes, and others), with variable training modalities (30–60% MIP) [70].

IMT (40% MIP, daily, 30 min per session, for 6 weeks) performed by HFrEF patients improves their functional motor capacity, balance, and muscle strength (improving their emotional status and dyspnea) [71].

About 40% of HF patients may suffer from an accidental fall within one year of their diagnosis [72]. In elderly subjects and those with comorbidities, the risk of death by falling in America is 24.9–69.4% [73].

Even if there are no sufficient statistical data or conclusive evidence to illustrate the reasons for such adjustments, including IMT in the therapeutic pathway of patients with HF may represent a very effective strategy.

## 5. Chronic HF Patients with Lower Back Pain

Over half of patients with HF, especially the elderly and women, have lumbar back pain. This slows down their active motor activity, such as physical activities and daily tasks, and can lead to further symptoms such as fatigue and depression [74]. Lower back pain has a negative impact on patients’ clinical conditions. Elderly women with lower back pain are at a higher risk of falling (50%) [75]. The diaphragm is a fundamental muscle for optimal posture and functioning of the dorso-lumbar area through the creation of thoraco-abdominal pressures. If it undergoes alterations in thickness and contraction capacity, it can cause back pain [76]. In patients with HF, the diaphragm is thinner and exhibits reduced contractile function compared to healthy individuals. Although a potential relationship between diaphragmatic dysfunction and lower back pain in this population has been hypothesized, current evidence is insufficient to draw definitive conclusions.

We know that IMT improves diaphragm function by 50% of the MIP and decreases perceived pain, thereby improving motor function, in young people [77,78]. Chronic lower back pain causes increased diaphragmatic fatigue with reduced proprioception. IMT in subjects without HF can increase postural control, improve proprioceptive management, and decrease perceived pain [79]. To the authors’ knowledge, there is a lack of robust data in the literature on the use of IMT and the possible functional or pain responses in patients with HF and lower back pain.

## 6. Additional Arguments for Including IMT

Improving functional diaphragmatic capacity increases thoracic rib movement, improving lung compliance, perfusion, and alveolar exchanges during respiratory acts in patients with HF [5,37]. A better-performing diaphragm helps reduce the intervention of the accessory inspiratory muscles, reducing the blood flow required by the accessory muscles; this translates into approximately 5–7% more blood delivered to the limbs [80].

Performing IMT has no side effects, and may be indicated for clinically stable, frail patients in the acute and chronic phase and in the presence of pulmonary hypertension [7,39,41].

By strengthening the diaphragm, the percentage of myocardial and cerebral ischemic events should be reduced [27].

One of the chronic comorbidities that accompanies patients with HF is chronic pain, which is seen in about 75–85%, and a lower EF value [81,82]. The causes are not always clear and can be linked to the presence of other chronic conditions (diabetes, COPD, tumors, osteoarthritis, and others), to age (the elderly), to the female sex, to changes in the patient’s perception of their symptoms, to intrinsic genetic variations, and to anxiety and depression [81,82,83]. Generally, chronic pain leads to diaphragm dysfunction, as in painful osteoarticular disorders, as well as abdominal and thoracic visceral disorders [84,85,86]. We know that a deep breath stimulates the parasympathetic response, raising the pain threshold in chronic and acute pathological situations [87,88,89,90]. Deep breathing involves the activation of the baroreflex (part of proprioception), which induces an increase in the pain threshold; the baroreflex itself is dysfunctional in HF patients [91,92]. IMT could increase the excursion of the diaphragm, allowing for deeper breaths and positively mitigating the subjective sensation of pain, thanks to the stimulation of the aortic and carotid baroreceptor area [92]. Probably, deep breaths allow patients to obtain a resonance between their heartbeat and their breath more quickly, better stimulating the baroreceptor areas [93]. We do not know if and with which parameters the use of IMT in HF patients would affect their chronic pain threshold.

## 7. Grey Areas and Research Directions

It is well known that constant physical activity creates specular and specific adaptations, both local and systemic. Patients with HF should engage in a lifelong physical activity program, not limited to the period of their hospitalization but continuing it independently following their discharge. The clinician should emphasize this concept and encourage the patient to make training a lifestyle.

As far as IMT is concerned, official recommendations are very scarce, not univocal, and not always present in guidelines or rehabilitation trials.

We know that routinely performed IMT in healthy elderly subjects (55–75% MIP, 30 breaths per day, 6 days per week) can bring about rapid improvements in their exercise tolerance, ventilatory efficiency, and general performance [94]. On the contrary, diaphragmatic fatigue during activities in healthy elderly subjects leads to a decline in the strength expressed by the limb muscles, decreased peripheral oxygenation and reduced general performance, with an increase in the sympathetic system [95]. In healthy subjects, engaging in IMT at 50% of their PImax induces diaphragmatic fatigue after approximately two minutes within a 20-min session that is structured as two-minute work periods followed by one-minute rest intervals. Electromyographic measurements have demonstrated a decline in diaphragmatic force output of up to 20%. The full recovery of diaphragmatic performance typically requires approximately 30 min of rest [96]. In another study, using a PImax of 60%, in healthy subjects, after 15 consecutive breaths, their diaphragmatic fatigue was recorded (measured by gastric twitch pressure and transdiaphragmatic twitch pressure) and was shown to decrease [97].

Moreover, women seem to have a higher threshold of diaphragmatic fatigue, although the systemic and local effects of such fatigue are equal in both sexes [98].

The diaphragm demonstrates training-induced adaptability across all age groups, further supporting the clinical utility of IMT. Like any skeletal muscle, this muscle is composed of different phenotypes (aerobic and anaerobic fibers), which enable different actions at rest and under stress. Training should take into account the presence of these different contractile fibers in order to restore respiratory functions at rest and other actions (e.g., swallowing and speech) and actions under stress (e.g., coughing, sneezing, and adjusting posture).

The American College of Sports Medicine recommends the following for stimulating the white fibers of skeletal muscle (in healthy subjects), which provide greater volume, strength, and coordination: a resistance level of 60–70% (45–50% for untrained individuals) of one’s 1RM (the maximum weight that can be lifted once), 8–12 repetitions (15 repetitions for untrained individuals), 2–4 sets per muscle group (1–3 sets for untrained individuals), and 2–3 sessions per week with 2–3 min of rest between sets. This should be ideal for increasing volume and strength. If the work volume is less strenuous than the previous session, a 2–10% increase can be used [99]. To stimulate aerobic fibers and engage the skeletal muscle endurance capacity of healthy subjects, the ACSM recommends using a load of 40–60% of the subjective 1RM, with 15–20 repetitions (10–15 for untrained individuals), and resting for a maximum of 90 s between sets (1 min for untrained individuals), with a frequency of 2–4 days per week (2–3 days for untrained individuals) [99].

PImax can be conceptually compared to 1RM, allowing for the application of the standard training principles used for targeting aerobic and anaerobic muscle fibers in untrained individuals. For anaerobic (white) fiber recruitment, a training intensity of 50–60% of the patient’s PImax (1RM equivalent) is appropriate, following a protocol of 1–3 sets of 15 repetitions with 2–3 min of rest between sets and not exceeding three sessions per week. For aerobic (red) fiber activation, a lower intensity of approximately 40% of the patient’s PImax could be used, with 10–15 repetitions, one minute of rest between sets, and a frequency of two to three sessions per week. Although the ACSM does not specify the number of sets for aerobic training, it is plausible to apply the same set range used for white fiber training to red fibers in this context.

This hypothesis could become a starting point for further research for this type of patient. In total, each daily session should have 2–6 series with a PImax/1RM of 40–60%, with the number of series dependent on the number of subjective sets, and a duration dependent on the training volume (which should be lower than that in trials reported in the literature), for 2–3 weekly sessions.

The concept of split training could be used for more delicate patients. This training model involves double daily training, but with different intensities and/or muscle groups involved, in healthy subjects [99]. The clinician could choose to recommend IMT for anaerobic fibers in the morning and IMT for aerobic fibers in the afternoon. Further investigations are necessary.

The diaphragm is involved in every aspect of our lives, from the simple acts of moving, breathing, and interacting with our environment to more intense training methods like ET, RT, and HIIT. Muscle adaptation to training stimuli occurs primarily during rest periods rather than the acute phase of exercise. Therefore, intense training protocols should incorporate longer rest intervals, especially considering that patients may also engage in additional exercise modalities such as cycling and resistance training (RT). Excessive training involving the diaphragm could result in a lack of positive adaptive responses (overtraining) [61]. If the patient with heart failure follows a demanding rehabilitation program, the intensity of the ET and RT processes should probably be modulated when IMT is also present.

## 8. Challenges for the Future

Despite cardiac rehabilitation being a non-invasive and low-cost approach, it is still underused. In countries with low technological, economic, and social development, access to this training program for patients with HF is extremely difficult; for one, there is no cardiac rehabilitation infrastructure, and additionally, the patient may not even have the rehabilitation tools to follow a rehabilitation program at home [100]. Some recent data on the percentage of patients with HF who follow rehabilitation programs are extremely worrying; the 2019 UK National Audit of Cardiac Rehabilitation (NACR) reported that less than 10% of patients can access or follow a rehabilitation program [100]. Similar data are found in America. The reasons for this are many and are not always clear. Clinicians should educate patients more on the importance of rehabilitation and encourage them to follow a training program at home, and policymakers should implement rehabilitation programs and enhance facilities [100]. The patients should understand clearly that rehabilitation training brings many benefits, which are lost if the recommended training program is not consistently followed. Rehabilitation should be carried out throughout their life. If a patient is economically active (employed), it might not be easy for them to integrate their work with the clinical center’s schedule. Additionally, laws might not grant them financial reimbursement [100].

As rehabilitation offers numerous local and systemic benefits, patients must be encouraged to follow training programs [101]. The challenges for the future of rehabilitation are not just in regards to patient education, but they concern society as a whole, from the clinician to the politician, and depend on the context in which care is provided. Facilities and access must be improved, economic aid must be provided to patients, the possibility of engaging in rehabilitation remotely (at home with technology) must be expanded, and other health professionals must be encouraged to undergo training to implement an interprofessional approach for patients who need rehabilitation [100,101].

Another challenge is to increase research on cardiac rehabilitation in the respiratory field, as we do not have data on the stratification and phenotypic differentiation of patients with HF. For example, emerging evidence highlights the cardiopulmonary interplay in obese HFpEF phenotypes and the potential modulatory role of GLP-1 receptor agonists, highlighting the need for more subjective rehabilitation approaches [102].

## 9. Conclusions

One of the non-pharmacological and non-surgical pillars that can support a patient in their clinical path is cardiovascular rehabilitation. All international guidelines agree on the importance of following rehabilitation, but not all cardiology organizations place the same emphasis on diaphragm training in this patient population. The article has reviewed the rationale for a greater emphasis on inspiratory muscle training in rehabilitation, while also putting forward some new ideas for the organization of diaphragm training. Further research efforts must be made to obtain more information on the benefits and use of inspiratory muscle training in patients with chronic HF.

## Figures and Tables

**Table 1:** Brief summary of the benefits of rehabilitation training. ET, endurance training; RT, resistance training; HFpEF, patients with a preserved left ventricular ejection fraction (LVEF) (HFpEF with a value ≥ 50%).

Benefits of Physical Activity in Patients with Heart Failure
Combining ET and RT improves VO2max by about 4–16% and chronotropic incompetence; a 6% increase in VO2max reduces all-cause mortality and hospitalization in patients with HF by 5–8% [6,25,27,32].
Constant training reduces the activity of the sympathetic system and improves the antioxidant capacity of the organism; reduces systemic inflammatory values; improves endothelial structure and function; improves the oxygen extraction capacity of the periphery (skeletal muscles) at rest and under stress; leads to greater cardiac electrical stability; and stimulates the coronary collateral network [3,6,24,32].
Glycemic levels improve and are better controlled [24].
Some cardiac contractility parameters improve, mainly in patients with HFpEF [32].

**Table 2:** Brief overview of the recommendations that the major world health organizations provide for patients with HF. The American Heart Association (AHA), the American College of Cardiology (ACC), the Heart Failure Society of America (HFSA), the European Society of Cardiology (ESC) guidelines for the management of HF (heart failure). HFrEF, patients with a reduced left ventricular ejection fraction (LVEF) (HFrEF with a value ≤40%), maximal inspiratory pressure (PImax), inspiratory muscle training (IMT).

Organizations and Guidelines	Indications on Diaphragm Training (IMT)	Reference
American guidelines (AHA/ACC/HFSA)	No recommended procedure to improve the functional capacity of the respiratory muscles	Ref. [21]
ESC guidelines	Indications are specific to HFrEF. A resistance of 30% of the patient’s PImax, reaching up to 60% of the patient’s subjective PImax. The resistance must be recalibrated approximately every week, with 3–5 sessions per week and for 20–30 min per session.	Ref. [28]

**Table 3:** Brief overview of local and systemic adaptations when inspiratory muscle training (IMT) is present in the rehabilitation process in patients with heart failure. HFpEF, patients with a preserved left ventricular ejection fraction (LVEF) (HFpEF with a value ≥ 50%); HFrEF, patients with a reduced LVEF (HFrEF with a value ≤ 40%), HIIT, high-intensity interval training; ET, endurance training; PImax, maximal inspiratory pressure; VO2peak, oxygen uptake during peak exercise; NT-pro-BNP, N-terminal pro-brain natriuretic peptide.

HF Patient Adaptations When IMT is Present in Rehabilitation	References
IMT increases PImax, VO2peak, and the minute ventilation/carbon dioxide production (VE/VCO2) slope; reduces the sense of dyspnea; increases the distance covered in the 6-min walk test (in patients with HFpEF); improves their quality of life; reduces lactate levels; improves ventilation efficiency, especially when combined with HIIT; improves the baroreceptor reflex, with an increase in peripheral perfusion phenomena when exerting effort, consequently reducing peripheral chemoreceptor responses; and increases lower limb muscle strength. IMT combined with ET reduces the values of NT-pro-BNP and C-reactive protein	[3,5,7,24,26,27,36]
Improvements in chronotropic, diastolic, and systolic blood pressure and saturation; increased walking distance; and increased inspiratory force, probably due to a more optimal modulation of the autonomic system (in patients with HFrEF)	[39]
Improves quality of life (in patients with HFrEF)	[40]
In acute hospitalized patients (HFrEF), IMT increases functionality, measured in terms of the distance covered in the 2-min walking test in meters	[41]
IMT for HFrEF induces several functional improvements, such as a more balanced autonomic expression, less dyspnea, increased diaphragm thickness, and better arterial response (such as tone and vasodilation)	[42]

**Table 4:** Brief summary of the pathological changes in the respiratory musculature.

Rationale for the Use of IMT: Counteracting Pathological Changes
The respiratory muscles are hypotrophic with a metabolic/phenotypic change towards a greater number of anaerobic (poorly functioning) fibers and reduced capillarization, which leads to a more anaerobic environment [43].
Peripheral information from skeletal musculature will be recorded by the central nervous system as constant fatigue, with an overexcitation of the sympathetic system, vasoconstriction, a decreased VO2peak, an increased heart rate, and intolerance to effort [43].
There seems to be a degeneration of the synaptic plate with partial denervation and a reduction in the force expression of 15–30%; in animal models, the contraction speed is reduced by 20–30%, which leads to a decrease in peak force (for example, when coughing or under stress) of 35–50% [44].
There is a decline in the number of cross-bridges, titin (shock absorber protein), myosin heavy chain (MHC), and the movement capacity of the same protein, with a decrease in myosin ATPase activity [44].
There seems to be an increase in red fibers compared to anaerobic fibers (with the opposite tendency in the limbs) [46].
There is an increase in connective and adipose tissue within the diaphragm, which is a sign of atrophy, with a decline in MIP (maximal inspiratory pressure) of about 30% [45].
The diaphragm moves with a reduced excursion and with an inverse relationship between the severity of symptoms and contractile capacity. The diaphragm presents with a decreased thickness [48].
